# The cost of adding rapid screening for diabetes, hypertension, and COVID-19 to COVID-19 vaccination queues in Johannesburg, South Africa

**DOI:** 10.1186/s12889-024-19253-8

**Published:** 2024-07-16

**Authors:** Sithabiso D. Masuku, Alana T Brennan, Beatrice Vetter, Francois Venter, Bukelwa Mtshazo, Simiso Sokhela, Nkuli Mashabane, Kekeletso Kao, Gesine Meyer-Rath

**Affiliations:** 1 Health Economics and Epidemiology Research Office, University of the Witwatersrand, Unit 2, 39 Empire Road, Parktown Johannesburg, Johannesburg, 2193 South Africa; 2 Department of Global Health, Boston University School of Public Health, Boston, MA USA; 3 Department of Epidemiology, Boston University School of Public Health, Boston, MA USA; 4 FIND, Geneva, Switzerland; 5 Wits Ezintsha, Faculty of Health Sciences, University of the Witwatersrand, Johannesburg, South Africa; 6 DSI-NRF Centre of Excellence in Epidemiological Modelling and Analysis (SACEMA), Stellenbosch University, Stellenbosch, South Africa

**Keywords:** Hypertension, Diabetes, COVID-19, Cost, Rapid screening, South Africa, Opportunistic screening

## Abstract

**Background:**

Non-communicable diseases (NCDs) are responsible for 51% of total mortality in South Africa, with a rising burden of hypertension (HTN) and diabetes mellitus (DM). Incorporating NCDs and COVID-19 screening into mass activities such as COVID-19 vaccination programs could offer significant long-term benefits for early detection interventions. However, there is limited knowledge of the associated costs and resources required. We evaluated the cost of integrating NCD screening and COVID-19 antigen rapid diagnostic testing (Ag-RDT) into a COVID-19 vaccination program.

**Methods:**

We conducted a prospective cost analysis at three public sector primary healthcare clinics and one academic hospital in Johannesburg, South Africa, conducting vaccinations. Participants were assessed for eligibility and recruited during May-Dec 2022. Costs were estimated from the provider perspective using a bottom-up micro-costing approach and reported in 2022 USD.

**Results:**

Of the 1,376 enrolled participants, 240 opted in to undergo a COVID-19 Ag-RDT, and none tested positive for COVID-19. 138 (10.1%) had elevated blood pressure, with 96 (70%) having no prior HTN diagnosis. 22 (1.6%) were screen-positive for DM, with 12 (55%) having no prior diagnosis. The median cost per person screened for NCDs was $1.70 (IQR: $1.38-$2.49), respectively. The average provider cost per person found to have elevated blood glucose levels and blood pressure was $157.99 and $25.19, respectively. Finding a potentially new case of DM and HTN was $289.65 and $36.21, respectively. For DM and DM + HTN screen-positive participants, diagnostic tests were the main cost driver, while staff costs were the main cost driver for DM- and HTN screen-negative and HTN screen-positive participants. The median cost per Ag-RDT was $5.95 (IQR: $5.55-$6.25), with costs driven mainly by test kit costs.

**Conclusions:**

We show the cost of finding potentially new cases of DM and HTN in a vaccine queue, which is an essential first step in understanding the feasibility and resource requirements for such initiatives. However, there is a need for comparative economic analyses that include linkage to care and retention data to fully understand this cost and determine whether opportunistic screening should be added to general mass health activities.

**Supplementary Information:**

The online version contains supplementary material available at 10.1186/s12889-024-19253-8.

## Introduction

In South Africa, deaths from major non-communicable diseases (NCDs) increased by 58.7% over 20 years (2008–2018) [1]. In 2018, cardiovascular diseases (CVD) accounted for 17.6% of overall deaths, cancers for 9.6%, diabetes for 5.9% and chronic respiratory diseases for 3% of total mortality [1]. South Africa has a high burden of hypertension (HTN) and diabetes mellitus (DM). The 2016 South African Demographic and Health Survey (SADHS) estimated the prevalence of HTN to be between 18.9% and 37.1% and that of DM between 4.5% and 11.4% [2]. The prevalence of these conditions is on the rise due to improvements in HIV treatment and lifespan of people living with HIV [3–5], as well as the increase in sedentary lifestyle and changes in diet. [6–8] According to South African official government statistics, DM was the second-highest natural cause of death between 2015 and 2017, accounting for 5.7% of deaths in 2017, and out-ranked only by tuberculosis. [9] Hypertensive-linked diseases were responsible for 4.5% of deaths and were the sixth leading cause of death in 2016 and 2017. [9]

Early diagnosis and treatment are important to prevent complications from HTN and DM. When left untreated, DM can give rise to a wide range of complications affecting both the micro- and macrovascular system, including a two-fold increased risk of heart attacks and stroke and an increased risk of HTN, which further amplifies the risk of adverse cardiovascular outcomes. [10, 11] While the diagnosis, treatment and management of underlying DM and HTN are generally affordable, the expenses associated with treating their complications can be substantial. [12] Screening can identify people at risk for these conditions and refer them to appropriate care. Our recent systematic literature review found only 3 studies that reported the costs of screening for DM or HTN in South Africa between 1995 and 2022. [13] Two reported the costs of screening for diabetic retinopathy and found the screening methods used to be cost-effective, [14, 15], while the third estimated that integrating NCD screening to existing HIV testing increased the cost per person screened but had the potential to efficiently utilise resources compared to stand-alone services. [16] Currently, the South African National Department of Health (NDOH) recommends that all participants should be assessed for HTN at their first visit to a primary health care facility (PHC) by taking two readings 1–2 min apart using a blood pressure cuff and, if need be, a third, confirmatory reading. For DM, the NDOH recommends assessment using a random finger prick glucose test at the first PHC visit for participants over 45 or overweight participants. [17]

Despite this clear guidance, DM and HTN are underdiagnosed in South Africa, with the 2016 SADHS estimating that 61% and 49% of DM and HTN, respectively, are undiagnosed. [2, 18–20]

One of the ways to address the NCD screening gap is to opportunistically leverage other healthcare activities directed at large audiences, such as the recent COVID-19 vaccination efforts during 2022. In our previous study, we effectively demonstrated the potential of using South Africa’s existing COVID-19 screening activities for opportunistic screening of HTN and DM. [21] However, no information exists regarding the costs of such an integration. The aim of this study was to evaluate the cost of integrating DM and HTN screening (NCD screening) and COVID-19 antigen rapid diagnostic testing (Ag-RDT) into COVID-19 vaccination. We estimated the cost per person screened as well as the cost per person found to have elevated blood glucose levels or blood pressure with or without a previous diagnosis at enrollment.

## Methods

### Study design and setting

We conducted a prospective cost analysis at three public sector PHCs in Johannesburg, Gauteng Province, South Africa, Yeoville Recreational Centre, Hillbrow Community Health Centre, and Clermont Clinic, as well as one academic hospital (Charlotte Maxeke Johannesburg Academic Hospital, CMJAH). The first two facilities serve participants residing in inner-city residential neighbourhoods, while CMJAH provides services to the entire province. These areas are characterized by high population density, unemployment, and poverty. Participants were recruited between 18 May and 16 December 2022.

### Study participants

People queuing for COVID-19 vaccination at the above-mentioned facilities were approached for study enrollment and consent. Participants had the flexibility to visit the screening table or tent either before or after their vaccination without losing their place in the queue. Participants approached before receiving their COVID-19 vaccine were screened for COVID-19 symptoms and were given the choice to opt into the RDT testing. Those who opted in, regardless of symptoms, had a nasopharyngeal swab taken on which a point-of-care COVID-19 Ag-RDT was performed. While waiting for the Ag-RDT results, the study staff collected information regarding co-morbidities, including known diagnoses of DM or HTN and other chronic conditions, and conducted NCD screening. Participants were adults aged 18 years and above who were able to read and had a mobile phone capable of receiving USSD, SMS or WhatsApp messaging. Participants who were unable to provide informed consent or deemed to be at significant risk of failing to comply with the provisions of the protocol, vulnerable populations, and personnel directly involved in conducting the study were excluded. For the COVID-19 Ag-RDT, we also excluded participants with any contraindications to nasopharyngeal sample collection.

Individuals whose initial blood pressure reading was elevated underwent a second blood pressure measurement after five minutes of seated rest. Those who were screen-positive, i.e., exhibited elevated blood glucose levels (random glucose > 11.1 mmol/L or fasting glucose > 7.0 mmol/L) and/or high blood pressure (defined as diastolic blood pressure > 90 mmHg and systolic blood pressure > 140 mmHg) were provided with a written referral detailing the outcomes of their DM and HTN screening. The study staff strongly urged individuals to confirm diagnosis at their local PHC. Those who did not meet these criteria were categorised as screen-negative. All participants with elevated blood glucose had blood drawn and sent to a laboratory to measure their HbA1c and plasma blood glucose.

Our methodology for including participants who self-reported DM in blood testing and including participants with self-reported HTN is supported by evidence from a prior study. [22] This study indicated that a considerable proportion of DM and HTN cases were either undiagnosed or not receiving adequate treatment. By not excluding these individuals, our aim was to accurately assess blood sugar control and blood pressure control within the entire spectrum of DM and HTN cases, thus ensuring a thorough evaluation of uncontrolled DM and HTN within the population.

This approach is consistent with established research precedents and highlights the necessity of regular screening and the improvement of management strategies in the context of diabetes care.

### Costing perspective and cost components

We estimated screening costs from the provider’s perspective using a bottom-up micro-costing approach involving identifying and costing every input necessary for delivering the intervention. We first calculated the exact amount of resources used for each client, including the amount of time spent on each activity by study nurses, consumables used, laboratory tests conducted, and equipment required. We then multiplied the resource usage by the cost of each resource from the same period and, finally, added up these costs to determine the overall cost of the intervention.

All resource prices were from 2022, with the exception of equipment purchased in 2021, for which prices were adjusted to 2022 South African Rand (ZAR) using International Monetary Fund inflation rates for South Africa. [23] Costs were then converted to 2022 USD using the average exchange rate for that year (US$1 = ZAR16.33942). [24]

### Method of cost measurements and sources of data

We determined the staff costs by using the actual salaries of the nurses involved in the study. The salaries were sourced from Ezintsha, the implementing partner on this study. We identified the nurse or nurses who worked with each participant and directly linked the time they spent with the patient to their salary. The nurses recorded each activity’s start and stop times in REDCap™, a secure web application for building and managing online surveys and databases. This enabled us to keep track of the time spent and calculate the staff costs accurately.

The laboratory test costs were incurred only for participants with high blood glucose levels and included plasma glucose and HbA1c tests, visit kits (pre-labelled blood tubes, sample collection materials, and blood collection materials), and a general laboratory fee. We obtained these costs from invoices provided by Ezintsha. We also sourced costs for Ag-RDT kits, consumables, and equipment from Ezintsha.

To calculate the average cost per person found, the total cost of all participants screened was divided by the total number of DM or HTN-screen-positive participants. For participants with a positive screening result but no previous diagnosis at enrollment, the total costs were divided by the number of these participants to obtain the average cost per “potentially new” person found.

## Results

The analysis was based on 1,376 participants who met the inclusion criteria, including 240 (17.4%) participants who had a COVID-19 RDT (Table 1). A total of 809 (59%) participants were recruited from Hillbrow Community Health Centre, 280 (20%) from Yeoville Recreational Centre, 203 (15%) from Charlotte Maxeke Johannesburg Academic Hospital, and 84 (6%) from Clermont Clinic. 54% of participants were female, with a median age for all participants of 38 [IQR: 30, 47]. The median body mass index (BMI) of participants was 25.7 kg/m^2^ [IQR: 21.8, 30.8]; 6% were categorised as severely obese, 22% as obese, and 26% as pre-obese. 50% of participants were unemployed. None of the participants who had an RDT tested positive for COVID-19.

**Table 1:** Characteristics and demographics of participants screened

	Male	Female	Total
	*n* = 638 (46%)	*n* = 738 (54%)	*N* = 1376
**Age (years)** (n, %)			
18–29	169 (26.5)	147 (19.9)	316 (23.0)
30–39	185 (29)	249 (33.7)	434 (32.5)
40–49	144 (22.6)	192 (26)	336 (24.4)
50–59	87 (13.6)	104 (14.1)	191 (13.9)
*≥* 60	53 (8.3)	46 (6.2)	99 (7.2)
**Age (median; IQR)**	37.0 (29.0, 47.0)	39.0 (31.3, 47)	38.0 (30.0, 47.0)
**BMI categories** (n, %)			
Underweight (< 18.5 kg/m^2^)	42 (6.6)	18 (2.4)	60 (4.4)
Normal (18.5–24.9 kg/m^2^)	392 (61.4)	182 (24.7)	574 (41.7)
Pre-obese (25.0–29.9 kg/m^2^)	149 (23.4)	205 (27.8)	354 (25.7)
Obese (30.0–39.9 kg/m^2^)	51 (8)	255 (34.6)	306 (22.2)
Severely Obese ( > = 40 kg/m^2^)	4 (0.6)	78 (10.6)	82 (6.0)
**Body Mass Index (median; IQR)**	22.8 (20.5, 26.2)	29.2 (24.6, 34.5)	25.7 (21.8, 30.8)
**Employment Status** (n, %)			
Employed	284 (44.5)	403 (54.6)	687 (49.9)
Unemployed	354 (55.5)	335 (45.4)	689 (50.1)
**Vaccinated prior to enrollment** (n, %)	430 (67.4)	550 (74.5)	980 (71.2)
**Smoking Status** (n, %)			
Current	280 (43.9)	104 (14.1)	384 (27.9)
Former	45 (7.1)	18 (2.5)	63 (4.6)
Never	313 (49.1)	616 (83.5)	929 (67.5)
**Site**			
Yeoville Recreational Centre	87 (13.6)	193 (14.1)	280 (20.3)
Hillbrow Community Health Centre	435 (68.2)	374 (27.2)	809 (58.8)
Clermont Clinic	42 (6.6)	42 (3.1)	84 (6.1)
Charlotte Maxeke Johannesburg Academic Hospital	74 (11.6)	129 (9.4)	203 (14.8)
**Previous diabetes diagnosis at enrollment**	16 (2.6)	28 (3.8)	44 (3.2)
**Previous hypertension diagnosis at enrollment**	55 (8.7)	117 (15.9)	172 (12.5)
**Previous diagnosis of hypertension and diabetes at enrollment**	11 (1.8)	17 (2.4)	28 (2.1)
**other co-morbid conditions at enrollment (self-reported)**			
HIV	60 (9.4)	152 (20.6)	212 (15.4)
Mental health	3 (0.5)	2 (0.3)	5 (0.4)
Cardiovascular disease	6 (0.9)	8 (1.1)	14 (1.0)
Asthma	4 (0.6)	7 (1.0)	11 (0.8)
**Outcomes (n (%)**			
Elevated blood glucose level indicative of diabetes	10 (1.6)	12 (1.7)	22 (1.6)
Elevated blood glucose level indicative of diabetes (new*)	8 (1.3)	4 (0.6)	12 (0.9)
Elevated blood pressure indicative of hypertension	75 (11.8)	63 (8.6)	138 (10.1)
Elevated blood pressure indicative of hypertension (new*)	61 (9.6)	35 (4.8)	96 (7.0)
Elevated blood glucose level and blood pressure indicative of diabetes and hypertension	4 (0.7)	1 (0.2)	5 (0.4)
Elevated blood glucose level and blood pressure indicative of diabetes and hypertension (new*)	4 (0.7)	0 (0)	4 (0.3)

* - Participants who did not report previous diagnosis at enrollment

A proportion of the study participants self-reported pre-existing medical conditions at the time of enrollment, with 172 individuals (12.5%) reporting a previous HTN diagnosis at enrollment, 44 (3.2%) reporting being previously diagnosed with DM, and 28 (2.1%) reporting a dual diagnosis of both DM and HTN prior to their enrollment in the study. Out of the total participants screened, 138 participants (10.1%) had elevated blood pressure during the study visit, of which 96 (7.0%) had no previous HTN diagnosis at enrollment. Additionally, 22 participants (1.6%) had elevated blood glucose levels, with 12 (54.5%) not reporting a previous DM diagnosis at enrollment. Among them, 4 (0.3%) participants, all males, had both elevated blood pressure and blood glucose levels, neither of which had been previously diagnosed. We found a total of 104 potentially new NCD screen-positive participants (with no previous diagnosis at enrollment), accounting for 67% of all screen-positive participants.

For NCD screening, participants who tested positive for both DM and HTN had the longest visits, with a median duration of 34.67 min [IQR: 33.67–35.47] (Table 2a). Participants who tested positive for DM alone had a median visit duration of 19.52 min [IQR: 16.28–26.04], while those who tested positive for HTN alone had a median duration of 14.38 min [IQR: 11.46–18.47]. Taking patient vaccination history, COVID-19 symptom screening and administering the Ag-RDT took a median time of 3.09 min (interquartile range [IQR]: 0.97–5.3) (Table 2b). Administering the Ag-RDT with the nasopharyngeal swab and preparing and reading the test kit had a median duration of 2.4 min (IQR: 0.18–18.47). (Mean times are in Additional files 1 and 2.)

**Table 2a:** NCD median screening staff time (minutes)

	Median (IQR)			
	Diabetes screen-positive	Hypertension screen positive	Diabetes & hypertension screen-positive	Screen-negative
Clinical history taking	0.63 (0.19, 1.92)	0.42 (0.17, 0.87)	0.22 (0.2, 0.28)	0.25 (0.12, 0.62)
Diabetes and hypertension screening	6.97 (5.33, 14.4)	11.10 (8.62, 14.46)	17.03 (17.03, 18.67)	4.47 (3.58, 6.03)
Blood collection	6.73 (4.43, 8.23)	7.61 (5.42, 9.12)	7.98 (7.61, 10.26)	-
Referral	3.15 (2.2, 4.74)	3.33 (2.38, 3.90)	7.65 (0.2, 0.28)	-
**Total**	**19.52 (16.28, 26.04)**	**14.38 (11.46, 18.47)**	**34.67 (33.67, 35.47)**	**4.9 (3.93, 6.81)**

**Table 2b:** COVID-19 median testing staff time (minutes)

	Median (IQR)
Vaccination history taking	0.18 (0.12, 0.3)
COVID-19 symptom screening	0.30 (0.21, 0.65)
COVID-19 testing	2.40 (0.18, 4.35)
**Total**	**3.09 (0.97, 5.3)**

Table 3 presents the median NCD screening per patient costs per outcome by cost category. (Mean costs are in Additional file 3). The main cost driver for all patient categories except DM and DM + HTN screen-positive participants was the staff costs, which ranged from 61% of total costs in participants that were screen-negative for HTN to 75% for HTN screen-positive participants. Diagnostic tests comprised 69% of total costs for DM screen-positive participants, 72% for potentially new DM cases, and 66% for DM + HTN-screen-positive participants. Three of the 10 DM screen-positive participants who had a prior diagnosis did not have any laboratory tests done. The median cost per person screened, regardless of outcome, was $1.70 [IQR: $1.38-$2.49]. The median cost for HTN screen-positive participants was $3.53 (IQR: $2.79-$4.62) and $3.53 (IQR: $2.83-$4.60) for participants who had not been previously diagnosed. In comparison, participants who were screen-positive for DM had a median cost of $29.62 (IQR: $28.18-$31.50), while participants with newly identified elevated blood glucose levels had the same median of $29.62 (IQR: $28.13-$31.36). For participants who were screen-positive for both conditions, the median cost was $31.57 (IQR: $31.43-$34.12). Please see Additional file 4 for the cost of NCD screening per patient per procedure and Additional file 5 for quantities and unit costs.

**Table 3:** Median cost of NCD screening per patient outcome (2022 USD)

Patient category (*n*)	Staff (USD, %)	Consumables (USD, %)	Diagnostic tests^2^ (USD, %)	Equipment (USD, %)	Overall median (USD, IQR)
All participants (1376)	1.60 (63)	0.61 (24)	0.31 (12)	0.01 (1)	1.70 (1.38, 2.49)
DM screen positive participants (22)	5.50 (20)	2.89 (11)	18.98 (69)	0.01 (0)	29.62 (28.18, 31.50)
DM screen positive participants – potentially new^1^ (12)	4.85 (17)	3.03 (11)	20.14 (72)	0.01 (0)	29.78 (28.18, 31.43)
DM screen negative participants (1354)	1.54 (73)	0.57 (27)	0.00 (0)	0.01 (1)	1.69 (1.38, 2.49)
HTN screen positive participants (138)	3.41 (75)	0.65 (14)	0.48 (10)	0.02 (0)	3.53 (2.79, 4.62)
HTN screen positive participants – potentially new^1^ (96)	3.41 (74)	0.66 (14)	0.50 (11)	0.02 (0)	3.53 (2.83, 4.60)
HTN screen negative participants (1248)	1.40 (61)	0.60 (26)	0.28 (12)	0.01 (1)	1.62 (1.36, 2.14)
DM + HTN screen positive participants (3)	7.94 (24)	3.25 (10)	21.98 (66)	0.02 (0)	31.57 (31.43, 34.12)
DM + HTN screen negative participants (1360)	1.35 (70)	0.57 (29)	0.00 (0)	0.01 (1)	1.62 (1.36, 2.11)

^*1*^ *Participants who did not report previous diagnosis at enrollment*

^*2*^ *Blood collection materials and lab handling fees*

Additional file 6 shows the cost of COVID-19 screening per patient by procedure. The median cost per Ag-RDT was $5.95 (IQR: $5.55-$6.25). The costs were driven mainly by the consumables, which accounted for approximately 90% of total costs. Please see Additional file 7 for COVID-19 screening quantities and unit costs.

The total cost of screening all 1,367 participants ($3,475.75) was divided by the prevalence of DM and HTN, combining previously diagnosed cases and potentially new cases, to estimate the average cost per person found. The average cost per person found to have elevated blood glucose was approximately $157.99, regardless of prior diagnosis, and $289.65 for potentially new cases. For elevated blood pressure, the estimated average cost per person found was $25.19, irrespective of prior diagnosis and $36.21 for potentially new cases. See Table 4.

**Table 4:** Cost per person found (2022 USD)

	Diabetes	Hypertension
Cost per potentially new* person found (n)	289.65 (12)	36.21 (96)
Cost per person found (n)	157.99 (22)	25.19 (138)
**Total cost (n)**	**3,475.78 (1,376)**	

*Participants who did not report previous diagnosis at enrollment

## Discussion

Opportunistically incorporating NCD screening into ongoing mass health interventions such as COVID-19 vaccination programs could offer significant long-term benefits in terms of early detection and initiation of early treatment of NCDs; however, it also poses challenges related to costs and resources. The aim of this study was to evaluate the cost of integrating NCD screening and COVID-19 Ag-RDT into COVID-19 vaccination. We estimated that integrating NCD screening into COVID-19 vaccination will cost $1.70 [IQR: 1.38–2.49] per person screened and $289.65 and $36.21 per potentially new case of DM and HTN, respectively. This is because only some of the participants (70%) were screen-positive for HTN with no previous diagnosis at enrollment, while 58% who were screen-positive for DM were unaware of their status. Cost drivers are dependent on the patient category. The main cost drivers for all patient types except DM and DM + HTN screen-positive participants were staff costs, accounting for between 61% and 75% of all costs. Diagnostic tests were the main cost driver for DM and DM + HTN screen-positive participants accounting for 72% and 66% of all costs, respectively. We estimated that the median cost per Ag-RDT was $5.95 (IQR: $5.55-$6.25) and its costs were driven mainly by consumables.

There is limited South African evidence on the cost of integrating NCD screening into routine care. Our systematic literature review which included papers on the costs of NCDs in South Africa published between 1995 and 2022 [13], only identified one study which reported the cost of integrating NCD screening to existing HIV testing, excluding point-of-care cholesterol testing, at an additional $2.24 per person screened. [16] This cost is comparable to our mean cost of $2.53 per person screened even though their study estimated the costs of an operational model as opposed to a research-related one and assumed task shifting to community care workers instead of nurses costed in our study.

The cost of finding a potentially new case of DM and HTN per person is notably high, primarily due to the lower prevalence of these conditions in our study population. In settings with higher prevalence, this cost would be lower. For instance, in our previous study conducted at taxi ranks in Johannesburg, we identified potentially new cases of DM in 5.2% of participants and potentially new cases of HTN in 19.3% [21], in contrast to the current study where these rates were only 0.9% and 7%, respectively.

To our knowledge, this is the first South African study that quantifies the cost of integrating NCD screening into mass health activities. The study uses participants who visit both primary and tertiary care public health facilities in Johannesburg, South Africa and provides cost estimates that may be applicable to other facilities in South Africa. There are, however, a number of limitations to our study. First, the costing was carried out in a study setting and not routine care. As such, it may not fully capture the scale, real-world conditions or complexity of routine clinical practice, and we may have overestimated routine care costs where we may have task shifting of screening to lower-level staff. However, the study setting ensured consistency in data collection, screening and measurement, reducing the risk of confounding factors and improving the reliability of our results.

Second, the study period is too short to quantify the impact of screening on health outcomes, such as linkage to care and retention, which would help determine the full cost-effectiveness of implementing general NCD screening. While a cost-outcomes analysis such as ours provides some indication for the cost per a specific outcome (here, per person screened), there is not enough data on the cost of routine NCD screening available for decision-making regarding whether the screening strategy is worthwhile in terms of health outcomes and resource allocation.

Lastly, our study has limited generalisability. The cost of screening can vary significantly based on factors such as the types of screening tests used, the professional levels of nurses carrying out screening, and the prevalence of NCD risk in the target population. Depending on these factors, screening costs in different settings may be higher or lower than we estimated. This may limit the generalisability of our findings to the context in which the study was conducted. However, our study helps us to understand the local resource requirements and lays the groundwork for the cost and cost-effectiveness of opportunistic NCD screening.

## Conclusions

We show the cost of finding potentially new cases of DM and HTN in a vaccine queue, which is an essential first step in understanding the feasibility and resource requirements for such initiatives. However, the decision to roll out opportunistic screening should be based on a comprehensive evaluation that compares the cost per person screened and per person screened positive for different screening modalities, including those integrated with other activities, such as successful linkage to care.

### Electronic supplementary material

Below is the link to the electronic supplementary material.

Supplementary Material 1

Supplementary Material 2

Supplementary Material 3

Supplementary Material 4

Supplementary Material 5

Supplementary Material 6

Supplementary Material 7

## Data Availability

All data generated or analysed during this study are included in this published article [and its supplementary information files].
