# Supplementary material for: The cost of adding rapid screening for diabetes, hypertension, and COVID-19 to COVID-19 vaccination queues in Johannesburg, South Africa
Source: BMC Public Health. 2024 Jul 16;24:1900. doi: 10.1186/s12889-024-19253-8 (PMC11251297; doi:10.1186/s12889-024-19253-8)
Supplement: Supplementary file 1 — Supplementary Material 1 [file 12889_2024_19253_MOESM1_ESM.docx]

**Table S1a:** **Mean NCD screening staff time (minutes)**

|  | **Mean time (SD)** | | | |
| --- | --- | --- | --- | --- |
|  | **Diabetes screen-positive** | **Hypertension screen-positive** | **Diabetes & hypertension screen-positive** | **Screen-negative** |
| Clinical history taking | 1.28 (1.40) | 0.87 (1.72) | 0.25 (0.09) | 0.61 (1.20) |
| Diabetes and hypertension screening | 9.38 (5.37) | 12.09 (5.51) | 18.12 (1.89) | 5.55 (3.63) |
| Blood collection | 6.73 (3.54) | 9.25 (5.18) | 9.25 (2.87) | - |
| Referral | 4.24 (3.01) | 3.52 (1.78) | 6.92 (3.71) | - |
| **Total** | **21.42 (8.42)** | **15.77 (7.11)** | **34.54 (1.81)** | **6.27 (4.48)** |
