# Supplementary material for: The cost of adding rapid screening for diabetes, hypertension, and COVID-19 to COVID-19 vaccination queues in Johannesburg, South Africa
Source: BMC Public Health. 2024 Jul 16;24:1900. doi: 10.1186/s12889-024-19253-8 (PMC11251297; doi:10.1186/s12889-024-19253-8)
Supplement: Supplementary file 2 — Supplementary Material 2 [file 12889_2024_19253_MOESM2_ESM.docx]

**Table S1b: Mean COVID-19 testing staff time (minutes)**

|  | **Mean time (SD)** |
| --- | --- |
| Vaccination history taking | 0.24 (0.17) |
| COVID-19 symptom screening | 0.69 (1.52) |
| COVID-19 testing | 3.02 (3.28) |
| **Total** | **3.87 (3.97)** |
