# Supplementary material for: The cost of adding rapid screening for diabetes, hypertension, and COVID-19 to COVID-19 vaccination queues in Johannesburg, South Africa
Source: BMC Public Health. 2024 Jul 16;24:1900. doi: 10.1186/s12889-024-19253-8 (PMC11251297; doi:10.1186/s12889-024-19253-8)
Supplement: Supplementary file 3 — Supplementary Material 3 [file 12889_2024_19253_MOESM3_ESM.docx]

**Table S2: Mean cost of NCD screening per patient by outcome (2022 USD)**

| **Patient category (n)** | **Staff** (USD, %) | **Consumables** (USD, %) | **Diagnostic tests^2^** (USD, %) | **Equipment** (USD, %) | **Overall mean** (SD) |
| --- | --- | --- | --- | --- | --- |
| All participants (1376) | 1.60 (63) | 0.61 (24) | 0.308 (12) | 0.01 (1) | 2.53 (3.62) |
| DM screen positive participants (22) | 5.50 (20) | 2.89 (11) | 18.98 (69) | 0.01 (0) | 27.38 (3.74) |
| DM screen positive participants – new^1^ (12) | 4.85 (17) | 3.03 (11) | 20.14 (72) | 0.01 (0) | 28.04 (8.11) |
| DM screen negative participants (1354) | 1.54 (73) | 0.57 (27) | 0.00 (0) | 0.01 (1) | 2.12 (1.23) |
| HTN screen positive participants (138) | 3.41 (75) | 0.65 (14) | 0.48 (10) | 0.02 (0) | 4.55 (4.58) |
| HTN screen positive participants – new^1^ (96) | 3.41 (74) | 0.66 (14) | 0.50 (11) | 0.02 (0) | 4.58 (4.68) |
| HTN screen negative participants (1248) | 1.40 (61) | 0.60 (26) | 0.28 (12) | 0.01 (1) | 2.30 (3.43) |
| DM+HTN screen positive participants (3) | 7.94 (24) | 3.25 (10) | 21.98 (66) | 0.02 (0) | 33.18 (3.03) |
| DM+HTN screen negative participants (1360) | 1.35 (70) | 0.57 (29) | 0.00 (0) | 0.01 (1) | 1.93 (1) |
