# Supplementary material for: The cost of adding rapid screening for diabetes, hypertension, and COVID-19 to COVID-19 vaccination queues in Johannesburg, South Africa
Source: BMC Public Health. 2024 Jul 16;24:1900. doi: 10.1186/s12889-024-19253-8 (PMC11251297; doi:10.1186/s12889-024-19253-8)
Supplement: Supplementary file 4 — Supplementary Material 4 [file 12889_2024_19253_MOESM4_ESM.docx]

**Table S3: Cost of NCD screening per patient per procedure (2022 USD)**

| **Clinical history taking** | Nurse | 0.14 (0.26) | 0.06 (0.03, 0.14) |
| --- | --- | --- | --- |
|  | **Total** | **0.14 (0.26)** | **0.06 (0.03, 0.14)** |
| **Diabetes and hypertension screening** | Nurse | 1.34 (1) | 0.99 (0.74, 1.56) |
|  | Consumables | 0.57 (0.01) | 0.56 (0.56, 0.56) |
|  | Equipment | 0.01 (0) | 0.01 (0.01, 0.01) |
|  | **Total** | **1.92 (1.00)** | **1.57 (1.32, 2.14)** |
| **Blood collection** | Nurse | 1.63 (1.17) | 1.17 (0.83, 2.50) |
|  | Consumables | 2.64 (0) | 2.64 (2.64, 2.64) |
|  | Diagnostic tests | 21.98 (0) | 21.98 (21.98, 21.98) |
|  | Equipment | 0 (0) | 0 (0, 0) |
|  | **Total** | **26.25 (1.17)** | **25.79 (25.45, 27.12)** |
| **Referral** | Nurse | 0.94 (1.37) | 0.7 (0.51, 1.04) |
|  | Consumables | 0.04 (0) | 0.04 (0.04, 0.04) |
|  | **Total** | **0.88 (0.5)** | **0.74 (0.55, 1.06)** |
| **Overall** | Nurse | 1.60 (1.38) | 1.12 (0.8, 1.89) |
|  | Consumables | 0.61 (0.31) | 0.56 (0.56, 0.56) |
|  | Diagnostic tests | 21.98 (0) | 21.98 (21.98, 21.98) |
|  | Equipment | 0.01 (0) | 0.01 (0.01, 0.01) |
|  | **Total** | **2.53 (3.62)** | **1.70 (1.38, 2.49)** |
