# Supplementary material for: The cost of adding rapid screening for diabetes, hypertension, and COVID-19 to COVID-19 vaccination queues in Johannesburg, South Africa
Source: BMC Public Health. 2024 Jul 16;24:1900. doi: 10.1186/s12889-024-19253-8 (PMC11251297; doi:10.1186/s12889-024-19253-8)
Supplement: Supplementary file 5 — Supplementary Material 5 [file 12889_2024_19253_MOESM5_ESM.docx]

**Table S4: Quantities and unit costs for NCD screening (2022 USD)**

|  | **Resource** | **Unit costs** | **Unit** | **Mean number of units per patient (SD)** |
| --- | --- | --- | --- | --- |
| **Clinical history taking** | Nurse time | 0.22 | minute | 0.64 (1.27) |
| **Diabetes and hypertension screening** | Nurse time | 0.22 | minute | 6.19 (4.29) |
|  | Sanitiser | 0.01 | ml | 4.00 (0) |
|  | Alcohol swabs | 0.01 | each | 1.00 (0.05) |
|  | Lancets | 0.06 | each | 1.00 (0.04) |
|  | Glucose strips | 0.24 | each | 1.00 (0.04) |
|  | Gloves | 0.10 | each | 1.98 (0.12) |
|  | Cotton wool balls | 0.01 | each | 1.00 (0.03) |
|  | Glucose meters | 0.003 | reading | 1.00 (0) |
|  | Sphygmomanometers | 0.003 | reading | 1.20 (0.4) |
|  | Scales with weight measuring rod | 0.01 | reading | 1.00 (0) |
| **Blood collection** | Nurse time | 0.24 | minute | 6.73 (3.54) |
|  | Sanitiser | 0.01 | ml | 6.00 (0) |
|  | Kit^1^ | 2.53 | kit | 1.00 (0) |
|  | HbA1c test | 15.50 | test | 1.00 (0) |
|  | Plasma glucose test | 5.06 | test | 1.00 (0) |
|  | General laboratory fee^2^ | 1.41 | sample | 1.00 (0) |
|  | Tourniquet | 0.0004 | blood draw | 1.00 (0) |
| **Referral** | Nurse time | 0.25 | minute | 3.85 (2.11) |
|  | Referral forms | 0.04 | each | 1.00 (0) |

^1^Contents of kit -includes pre-labelled blood tubes, sample collection materials and blood collection materials

^2^ Includes general sample receipt and handling, data clarification form, data management and project management per participant visit
