# Supplementary material for: The cost of adding rapid screening for diabetes, hypertension, and COVID-19 to COVID-19 vaccination queues in Johannesburg, South Africa
Source: BMC Public Health. 2024 Jul 16;24:1900. doi: 10.1186/s12889-024-19253-8 (PMC11251297; doi:10.1186/s12889-024-19253-8)
Supplement: Supplementary file 6 — Supplementary Material 6 [file 12889_2024_19253_MOESM6_ESM.docx]

**Table S5: Cost of COVID-19 screening per patient by procedure (2022 USD)**

|  | **Resource, % of total costs** | **Mean (SD)** | **Median (IQR)** |
| --- | --- | --- | --- |
| **Vaccination history taking** | Nurse time, 100% | 0.06 (0.06) | 0.04 (0.02, 0.08) |
|  | **Total** | **0.06 (0.06)** | **0.04 (0.02, 0.08)** |
| **COVID-19 symptom screening** | Nurse time, 100% | 0.16 (0.38) | 0.08 (0.04, 0.14) |
|  | **Total** | **0.16 (0.38)** | **0.08 (0.04, 0.14)** |
| **COVID-19 testing** | Nurse time, 10% | 0.63 (0.86) | 0.46 (0.06, 0.76) |
|  | Consumables, 90% | 5.50 (0.17) | 5.49 (5.49, 5.49) |
|  | **Total** | **6.13 (0.87)** | **5.95 (5.55, 6.25)** |
