# Supplementary material for: The cost of adding rapid screening for diabetes, hypertension, and COVID-19 to COVID-19 vaccination queues in Johannesburg, South Africa
Source: BMC Public Health. 2024 Jul 16;24:1900. doi: 10.1186/s12889-024-19253-8 (PMC11251297; doi:10.1186/s12889-024-19253-8)
Supplement: Supplementary file 7 — Supplementary Material 7 [file 12889_2024_19253_MOESM7_ESM.docx]

**Table S6: Quantities and unit costs for COVID-19 screening (2022 USD)**

|  | **Resource** | **Unit costs** | **Unit** | **Mean number of units per patient (SD)** |
| --- | --- | --- | --- | --- |
| **Vaccination history taking** | Nurse time | 0.24 | Minutes | 0.28 (0.29) |
| **COVID-19 symptom screening** | Nurse time | 0.23 | Minutes | 0.69 (1.52) |
| **COVID-19 testing** | Nurse time | 0.21 | Minutes | 3.01 (3.28) |
|  | Sanitiser | 0.01 | ml | 6.00 (0) |
|  | Kit^1^ | 2.60 | kit | 1.00 (0) |
|  | Disposable protective gown | 2.57 | gown | 1.00 (0) |
|  | Mask | 0.05 | mask | 1.00 (0) |
|  | Gloves | 0.10 | glove | 2.00 (0) |

^1^All Visit Kit - includes sample collection materials
